# Further thermo‐stabilization of thermophilic rhodopsin from *Thermus thermophilus* JL‐18 through engineering in extramembrane regions

**DOI:** 10.1002/prot.26015

**Published:** 2020-10-28

**Authors:** Tomoki Akiyama, Naoki Kunishima, Sayaka Nemoto, Kazuki Kazama, Masako Hirose, Yuki Sudo, Yoshinori Matsuura, Hisashi Naitow, Takeshi Murata

**Affiliations:** ^1^ RIKEN RSC‐Rigaku Collaboration Center RIKEN SPring‐8 Center Sayo‐gun Hyogo Japan; ^2^ RIKEN SPring‐8 Center Sayo‐gun Hyogo Japan; ^3^ Department of Chemistry, Graduate School of Science, and Molecular Chirality Research Chiba University Chiba Japan; ^4^ Malvern Panalytical division of Spectris Co., Ltd Tokyo Japan; ^5^ Division of Pharmaceutical Sciences, Graduate School of Medicine, Dentistry, and Pharmaceutical Sciences Okayama University Okayama Japan

**Keywords:** differential scanning calorimetry, membrane protein, molecular dynamics, optogenetics, protein stability, site‐directed mutagenesis

## Abstract

It is known that a hyperthermostable protein tolerable at temperatures over 100°C can be designed from a soluble globular protein by introducing mutations. To expand the applicability of this technology to membrane proteins, here we report a further thermo‐stabilization of the thermophilic rhodopsin from *Thermus thermophilus* JL‐18 as a model membrane protein. Ten single mutations in the extramembrane regions were designed based on a computational prediction of folding free‐energy differences upon mutation. Experimental characterizations using the UV‐visible spectroscopy and the differential scanning calorimetry revealed that four of ten mutations were thermo‐stabilizing: V79K, T114D, A115P, and A116E. The mutation‐structure relationship of the TR constructs was analyzed using molecular dynamics simulations at 300 K and at 1800 K that aimed simulating structures in the native and in the random‐coil states, respectively. The native‐state simulation exhibited an ion‐pair formation of the stabilizing V79K mutant as it was designed, and suggested a mutation‐induced structural change of the most stabilizing T114D mutant. On the other hand, the random‐coil‐state simulation revealed a higher structural fluctuation of the destabilizing mutant S8D when compared to the wild type, suggesting that the higher entropy in the random‐coil state deteriorated the thermal stability. The present thermo‐stabilization design in the extramembrane regions based on the free‐energy calculation and the subsequent evaluation by the molecular dynamics may be useful to improve the production of membrane proteins for structural studies.

## 1 INTRODUCTION

To prepare protein samples in structural biology, a thermostable mutant is often effective especially in the case of membrane proteins including G protein‐coupled receptors,^1^, ^2^, ^3^ AMPA receptor,^4^ purine/H^+^ symporter,^5^ and integral membrane diacylglycerol kinase.^6^ Currently, the establishment of such thermostable mutants requires a laborious screening in many cases. Therefore, a rational design of thermostable mutants is a desirable technology in the structural biology of membrane proteins. Recently, a computational method has been developed to identify possible thermo‐stabilizing mutations in the intramembrane region of a membrane protein.^7^, ^8^ However, there is no report to date for the rational thermo‐stabilization design in the extramembrane regions.

It is known that certain thermophilic organisms have “hyperthermostable” proteins tolerable at temperatures over 100°C.^9^, ^10^ Recently, we reported a rational hyperthermostabilization design of a soluble globular protein, CutA1, based on a computational prediction of folding free‐energy differences upon a site‐directed mutagenesis.^11^ In the case of CutA1, the denaturation temperature was improved from 86°C to 137°C by introducing two hydrophobic and six ionic mutations; both the enthalpic effect of hydrophobic interactions and the entropic/enthalpic effect of electrostatic interactions contributed to the stabilization.

In this work, the same strategy as that used for CutA1 has been applied to a thermostable membrane protein, toward the design of hyperthermostable mutants. As a model membrane protein, we selected the thermophilic rhodopsin from *Thermus thermophilus* JL‐18 (TR) that had been discovered as the first rhodopsin from thermophilic organisms.^12^ The crystal structure of TR is available (PDB entry 5AZD),^13^ indicating that the effect of mutation can be evaluated by the molecular dynamics (MD) simulation. Because the hyperthermostabilization strategy was originally developed on a soluble globular protein, we applied it in the extramembrane regions of TR. TR is composed of 260 amino‐acid residues with seven‐transmembrane helices, and functions as a proton pump in response to the light where a covalently‐bound retinal is used as a sensing cofactor. By utilizing its high thermostability, TR is expected as a useful tool for the optogenetics that controls an organism by the light.^13^, ^14^ Therefore, the hyperthermostable TR should expand its applicability as a thermostable biosensor in biotechnology. Here we report a rational design of further thermostable TR mutants through engineering in the extramembrane regions.

## 2 METHODS

### 2.1 Design of mutants

**FIGURE 1:**
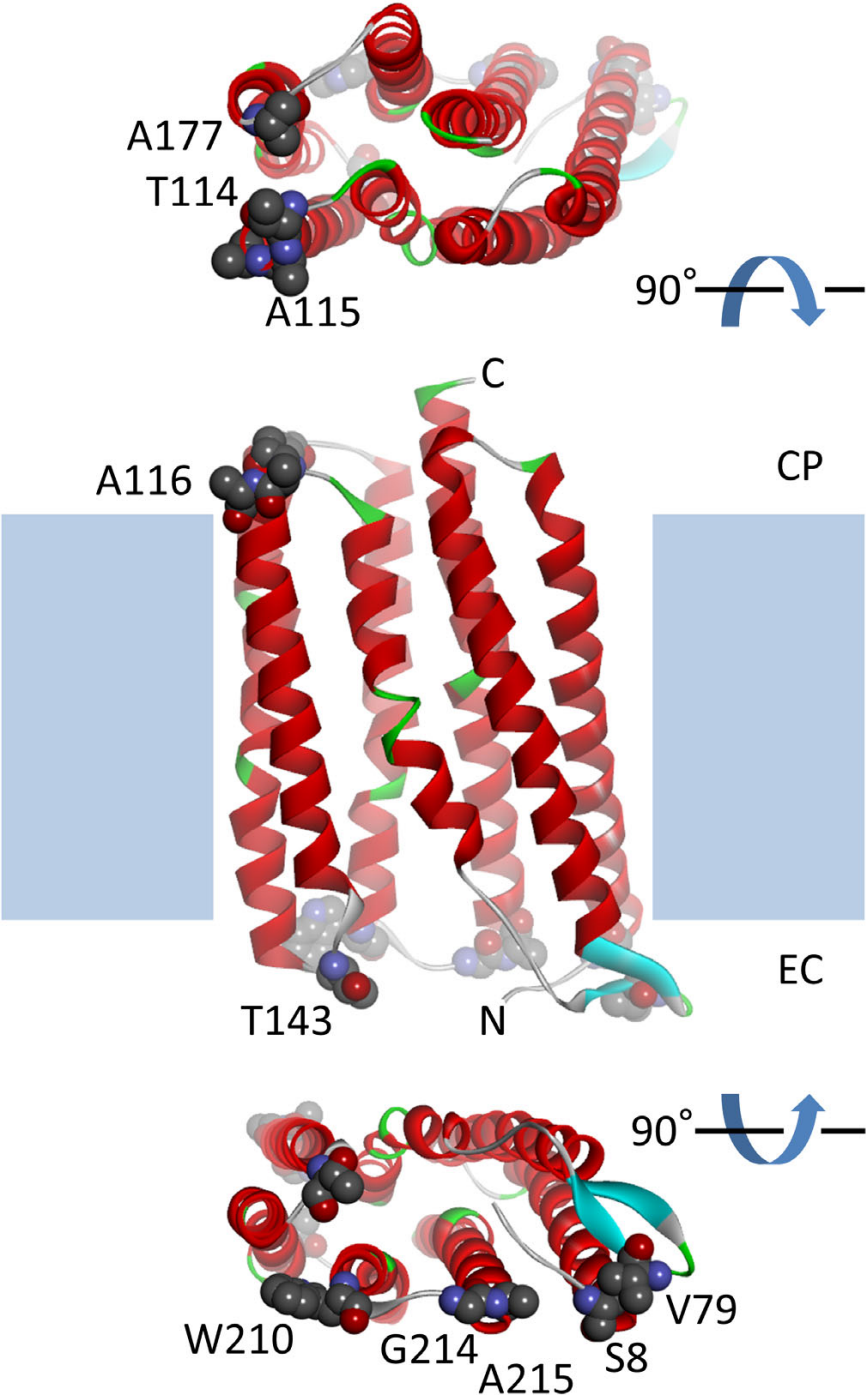
Location of 10 residues for mutation. The TR model is shown as a ribbon representation with secondary‐structure colorings: red, light blue, and green represent helix, sheet, and turn, respectively. The N‐ and C‐termini are labeled. The model is embedded in a lipid bilayer membrane depicted as blue squares; the characters CP and EC mean cytoplasmic and extracellular sides of the cell, respectively. The 10 amino‐acid residues are shown as space‐filling models with labels and atom‐type colorings. This figure was produced using the software *Discovery Studio* [Color figure can be viewed at wileyonlinelibrary.com]

The protein model of TR (UniProtKB H9ZSC3) used was prepared using the “Clean Protein” function of the software *Discovery Studio* v4.1 (BIOVIA; https://www.3dsbiovia.com/) from the chain A of the PDB entry 5AZD (amino acids 3‐253)^13^: missing side chains at the residues 89‐91, 178, and 253 were mended considering rotamer conformations; the residue 253 was modeled as a C‐terminus by attaching the OXT atom. Thermo‐stabilizing mutants of TR were designed using the program *FoldX* (http://foldxsuite.crg.eu/).^15^ Before the design, the retinal group attached to Lys233 in the TR model was removed, and then the model was treated by the “Repair PDB” function of *FoldX*. Twenty‐eight solvent‐exposed residues in the extramembrane region were selected manually using *Discovery Studio*: Ser8(N), Phe9(N), Ala41(C), Gly71(N), Gln74(N), Leu75(N), Gln76(N), Val79(N), Val81(N), Thr83(N), Pro86(N), Thr114(C), Ala115(C), Ala116(C), Asn142(N), Thr143(N), Gly146(N), Ala177(C), Gly180(C), Arg182(C), Trp210(N), Pro212(N), Gly214(N), Ala215(N), Ala216(N), Leu249(C), Glu250(C), Glu251(C); characters in parentheses indicate the location of residues where N and C represent the N‐terminal and the C‐terminal sides of the protein, respectively. In the selection, a residue was excluded in case that: its main‐chain dihedral angle was allowed only for limited residue types; it was a proline or an acidic residue for capping an α helix; it was a basic residue at the C‐terminus of an *α* helix; it was an ionic residue making ion‐pairs with other residues; a native‐state MD simulation predicted a burial of the residue in the protein molecule or in the membrane. The 28 residues were submitted to the calculation of the folding free‐energy using the “Position Scan” function of *FoldX*. Twenty candidates of single mutations of high scores from the Position Scan were selected and were further analyzed using the “Build Model” function of *FoldX*: S8D(−2.30), Q76R(−0.48), V79K(−0.41), V81R(−0.26), T114D(−3.08), T114E(−2.77), A115P(−1.26), A116D(−2.12), A116E(−1.53), N142R(−0.70), T143K(−1.01), A177K(−1.24), G180P(−1.87), W210R(−0.50), G214D(−3.43), G214E(−1.43), A215P(−1.48), A216E(−1.28), L249R(−0.15), E250M(−1.30); values in parentheses represent the total folding‐free‐energy differences upon mutation in kcal/mol from the Position Scan. Based on the Build Model score, and taking the variety of suggested effects and the balance of the N‐ and C‐terminal molecular polarity into account, 10 final candidates for the thermo‐stabilizing mutations were selected (Figure 1 and Table 1); a mutant was excluded in case that a substantial structural change upon mutation was suggested by the Build Model.

**TABLE 1:** Ten selected candidates of thermo‐stabilizing mutants

Mutant^a^	ΔΔG (kcal/mol)^b^	Putative effects^c^
S8D (N)	−2.32	Helix dipole (−2.40)
V79K (N)	−0.45	Electrostatic (−0.21)
T114D (C)	−3.91	Helix dipole (−2.61)
A115P (C)	−1.32	Entropic (−1.08)
A116E (C)	−2.20	Helix dipole (−1.01)
T143K (N)	−1.02	Hydrogen bond (−1.76)
A177K (C)	−1.08	Helix dipole (−0.60)
W210R (N)	−0.66	Helix dipole (−0.33)
G214D (N)	−3.42	Helix dipole (−2.48)
A215P (N)	−1.54	Entropic (−1.12)

^a^ Characters in parentheses indicate the location of residues where N and C represent the N‐terminal and the C‐terminal sides of the protein, respectively.

^b^ Total folding‐free‐energy differences upon mutation from the “Build Model” function of *FoldX*.

^c^ Suggestions from *FoldX* with their contributions to the folding free‐energy differences in parentheses.

### 2.2 Sample preparation

The gene of TR, which was optimized to *Escherichia coli* codon‐usage, was inserted into the pET21c expression vector (Novagen, Madison, WI). The mutations were introduced by the site‐directed polymerase chain reaction mutagenesis using the PrimeSTAR Max DNA polymerase (TaKaRa). For the protein expression, transformed *E. coli* C43(DE3) cells harboring the plasmid were initially grown at 37°C in 10 mL of LB medium supplemented with ampicillin (the final concentration was 50 *μ*g/mL) and were directly inoculated into 1 L of LB medium containing ampicillin. The cells were grown in a rotary shaker at 30°C after 2.5 hours incubation with 0.5 mM isopropyl *β*‐D‐1‐thiogalactopyranoside (Wako, co. Ltd.) and with 10 *μ*M all‐*trans*‐retinal (Sigma). The cells were harvested by centrifugation at 4°C, resuspended in buffer‐A (50 mM Tris‐HCl and 1 M NaCl; pH 7.5), and disrupted by sonication. The cell debris was removed by centrifugation (9400 *g*, 10 minutes, 4°C). Crude membranes were collected by centrifugation (137 400 *g*, 45 minutes, 4°C) and washed with buffer‐A. For solubilization of the membranes, 1.5% *n*‐dodecyl *β*‐D‐maltoside (DDM; Anatrace, Maumee) was added, and the suspension was incubated for 30 minutes at 4°C. The solubilized membranes were isolated by high speed centrifugation (137 400 *g*, 45 minutes, 4°C), and the supernatant was loaded onto a 2 mL Ni Sepharose 6 Fast Flow column (GE Healthcare, Little Chalfont, UK) equilibrated with buffer‐B (50 mM Tris‐HCl, 1 M NaCl, and 0.1% DDM; pH 7.5) containing 40 mM imidazole. The bound samples were eluted with buffer‐B containing 300 mM imidazole. The partially purified samples were diluted to be an absorbance of 0.6 at the maximum absorption wavelength of 530 nm, and were used for the measurement of the residual pigment as described in the following paragraph. The samples were further purified by the size‐exclusion chromatography using a Superdex‐200 column (GE Healthcare) equilibrated with buffer‐C (50 mM Tris‐HCl, and 0.1% DDM; pH 7.5) after incubations at 70°C for 20 minutes and at 80°C for 10 minutes so that TR trimers were dissociated into monomers as described previously.^16^ The purified samples were diluted to be 0.5‐0.8 mg/mL and were stored at 4°C until use.

### 2.3 Measurement of thermostability

Unless otherwise noted, data was represented as mean ± SE of the mean (s.e.m.) from three independent experiments. To compare the thermostability of wild‐type and the mutants of TR, the residual pigment after incubation at 90°C for 4 minutes of the partially purified samples was measured at maximum absorption wavelength of 530 nm by the UV‐visible spectroscopy. Next, the residual pigment values at 530 nm of the purified samples in buffer‐C were plotted against time for the incubation at 90°C as described previously.^12^ The data was analyzed by a single exponential decay function to estimate a decoloration rate constant. We also determined the apparent midpoint temperature of denaturation (*T*_m_) of the purified samples by the differential scanning calorimetry (DSC) as described elsewhere.^17^ The DSC experiments in buffer‐C were performed on a MicroCal PEAQ‐DSC Automated (Malvern Panalytical, Malvern, United Kingdom) ramping from 30°C to 130°C at a scan rate of 1°C/min, for the wild‐type and the mutants of TR. The data was processed employing the MicroCal PEAQ‐DSC Analysis Software (Malvern Panalytical, Malvern, United Kingdom). The signal of a buffer reference measured under identical conditions was subtracted from the signal of the protein samples, the data were normalized to the molar protein concentration, and an interpolated baseline was subtracted. The *T*_m_ value was determined at the peak top of a heat capacity curve. For each construct, three independent experiments were performed to obtain the average *T*_m_ value. To evaluate the statistical significance between a pair of average *T*_m_ values from different constructs, a two‐tailed *t*‐test on averages was applied against the null hypothesis where the averages were the same; for each construct, the accordance with a normal distribution was confirmed at the significance level of 5% using the Kolmogorov‐Smirnov test. The equality of valiances was examined at the significance level of 5% using the *F*‐test. The Student's *t* test and the Welch's *t* test were adopted in the cases of equal and unequal variances, respectively.

### 2.4 Molecular dynamics calculations

The MD simulation of TR was performed using the program *GROMACS* ^18^ v5.0.5, with the CHARMM36 force field.^19^ Unless otherwise noted, the parameter was set to the default value in *GROMACS*. The chain A of 5AZD was used to prepare the protein model for the wild‐type TR. To prepare a mutant model, the residue to be mutated was replaced by a designated residue type with a rotamer conformation selected so as to provide favorable intramolecular interactions using the “Build and Edit Protein” function of the software *Discovery Studio* v4.1. The protein model was embedded into an implicit membrane with 40 Å thickness using the “Create and Edit Membrane” function and the TR‐membrane orientation was manually adjusted in *Discovery Studio* so that the TR helices were aligned perpendicular to the membrane. This protein model of TR was submitted to the “Membrane Builder” tool of the CHARMM‐GUI Input Generator (http://www.charmm-gui.org/)^20^ to prepare the TR system for the MD run adopting a periodic boundary condition with explicit membrane/solvent molecules: Glu106 was protonated according to the literature^13^; Lys233 was modified with a retinal group; the orientation of the input model was kept; the system was neutralized by adding Na^+^ and Cl^−^ ions to reach the salt concentration of 0.15 M; the equilibration temperature was set at 300 K. For instance, the TR system for the wild‐type TR had a rectangular unit‐cell with dimensions of *a* = *b* = 86.0 Å, and *c* = 122.8 Å which were similar to those from the literature^13^ and consisted of 84 927 atoms in total: 4 088 protein atoms, 25 326 POPC (1‐palmitoyl‐2‐oleoyl‐*sn*‐glycero‐3‐phosphatidylcholine) lipid atoms, 56 Na^+^ ions, 56 Cl^−^ ions, 55 401 TIP3P water^21^ atoms.

For the MD simulation of the TR system in the native state, first the system was equilibrated in an NPT ensemble at 300 K using *GROMACS*, according to the CHARMM‐GUI procedure: a steepest‐descents energy minimization with a tolerance of 1000 kJ /mol/nm; the first equilibration for 25 ps at 300 K with a random seed of initial velocity, a time step of 1 fs, the LINCS algorithm to constrain bonds involving hydrogen atoms,^22^ the particle‐mesh Ewald (PME) method for the electrostatic calculation,^23^ and with a temperature coupling according to Berendsen^24^; the second equilibration for 25 ps in the same way but with decreased geometric restraints; the third equilibration for 25 ps in the same way but with a pressure coupling at 1.0 bar according to Berendsen^24^ and with decreased restraints; the fourth equilibration for 100 ps in the same way but with a time step of 2 fs and with decreased restraints; the fifth/sixth equilibration for 100 ps in the same way but with decreased restraints. Then, an NPT simulation at 300 K for 100 ns was performed according to the CHARMM‐GUI protocol adopting a time step of 2 fs, the LINCS constraints, the PME electrostatics, the Nosé–Hoover thermostat,^25^ and the Parrinello–Rahman barostat^26^ at 1.0 bar; no geometric restraints was applied, the number of steps between the neighboring energy calculations (ie, nstcalcenergy) was set as 1 000 steps, the linear mode was selected as the center of mass motion removal at every 1 000 steps. The calculation performance was 2.3 to 6.2 ns per day using a Linux PC with 11 to 30 threads. The native‐state simulation was repeated for three or four times. For the MD simulation in the random‐coil state, the TR system equilibrated at 300 K in the same way as that in the native‐state simulation was submitted to an NVT simulation at 1 800 K for 1 ns with a time step of 1 fs, using the Berendsen thermostat, the LINCS constraints, and the PME electrostatics; no geometric restraints was applied, the nstcalcenergy was set as 200 steps; the linear mode was selected as the center of mass motion removal at every 200 steps. Since this high‐temperature simulation could be executed within a half day using a Linux PC with 11 to 30 threads, 20 independent runs were performed for the statistical evaluation. The NPT equilibration at 300 K was performed with different initial velocity before each NVT run at 1 800 K, to avoid a complete reproduction.

### 2.5 Analysis of structures

For the evaluation of the native‐state MD structures of a TR construct, 51 structures in the MD simulation at every 200 ps from 90 ns to 100 ns were fitted to the input model (ie, crystal structure) at corresponding C^*α*^ atoms using the “gmx rms” function of *GROMACS*, and the average root‐mean‐square deviation (r.m.s.d.) value from the 51 C^*α*^ superpositions was used for the statistical test. Each construct had three or four independent MD runs (*n* = 4 for T143K; *n* = 3 for the others) and therefore had three or four average r.m.s.d. values as a group. The random‐coil‐state MD structures of a TR construct were evaluated as follows: (a) C^*α*^ superpositions were performed within 20 random‐coil MD structures after the 20 independent MD runs at 1 800 K for 1 ns; (b) r.m.s.d. values from 19 superpositions between a selected MD structure and the others were calculated and averaged; (c) a representative MD structure was selected so as to give the smallest average r.m.s.d. value; (d) the r.m.s.d. values from the C^*α*^ superpositions between the representative structure and the remaining 19 structures were used for the statistical test. This group of 19 r.m.s.d. values represent the degree of an intramolecular structural variation and therefore is assumed to reflect the intramolecular structural fluctuation of a TR construct in the random‐coil state. For the statistical test, a two‐tailed Student's *t* test on averages was applied to compare a pair of r.m.s.d. groups from different constructs at the significance level of 5% against the null hypothesis where the averages were the same; for each group, the accordance with a normal distribution and the equality of valiances were confirmed at the significance level of 5% using the Kolmogorov–Smirnov test and the *F*‐test, respectively.

## 3 RESULTS

### 3.1 Selection of candidates for thermo‐stabilizing mutants

From 28 solvent‐exposed residues in the extramembrane regions, we designed ten thermo‐stabilizing mutants of TR based on the folding free‐energy calculation using the program *FoldX* (Figure 1 and Table 1). Of these single mutations, six and four sites were on the N‐ and C‐terminal side of the protein, respectively. The *FoldX* calculation suggested potential stabilizations due to various putative effects including six helix dipole effects, two main‐chain entropic effects, an electrostatic effect, and a hydrogen‐bond effect. The total folding free‐energy differences upon mutation were in the range of −3.91 to −0.45 kcal/mol. Probably reflecting the helical‐bundle architecture of the TR molecule, helix‐related suggestions were predominant: a helix‐dipole stabilization in the mutants S8D, T114D, A116E, and G214D that located at the N‐termini of helices, the same stabilization in the mutants A177K and W210R that located at the C‐termini of helices, and a stabilization due to the main‐chain entropy in the mutants A115P and A215P that located at the N‐termini of helices. The other suggestions were stabilization in the V79K mutant due to an ion‐pair formation between the side chains of Glu6 and Lys79, and a stabilization in the T143K mutant due to a hydrogen‐bond formation between the main‐chain oxygen of Val141 and the *ε*‐amino group of Lys143.

### 3.2 Thermostability of TR

**FIGURE 2:**
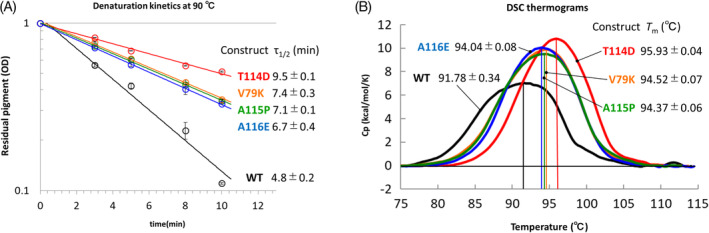
Experimental characterization of TR. A, Decoloration kinetics at 90°C of wild‐type and stabilized mutants of TR. Decay plots for the TR constructs are shown with values of τ_1/2_ representing the time after which half the initial sample has decayed. See Methods for details. B, DSC thermograms of wild‐type TR and stabilized mutants. Major endothermic transitions observed from the purified TR constructs in detergent micelle are shown with *T*_m_ values [Color figure can be viewed at wileyonlinelibrary.com]

We purified the wild‐type and 10 single mutants (S8D, V79K, T114D, A115P, A116E, T143K, A177K, W210R, G214D, and A215P) of TR. The UV‐visible spectroscopy of these purified samples exhibited the maximum absorption at the same wavelength, 530 nm. It is generally accepted for the rhodopsin family that the maximum absorption is strongly correlated with the protein properties.^12^, ^13^, ^14^ Therefore, to compare the thermostability of wild‐type and the 10 mutants of TR, a thermal‐decoloration of pigment at 530 nm after incubation at 90°C for 4 minutes was quantified (Table 2). As a result, the residual pigment values of four mutants (V79K, T114D, A115P, and A116E) were significantly higher than that of the wild type, suggesting a mutation‐induced thermo‐stabilization, whereas the other six mutants were suggested to be destabilized. The four potentially stabilized mutants were characterized further. A kinetics study using the UV–visible spectroscopy at 530 nm showed that the thermal decoloration was significantly slower in the four mutants when compared to the wild type (Figure 2A and Table 2). Another characterization by DSC showed the higher *T*_m_ values of the four mutants when compared to the wild type (*P* < .01; Figure 2B and Table 2), although these are apparent values because the denaturation of TR was an irreversible/kinetic process in the DSC experiment (Supplementary Figure S1). These two approaches (decaloration and DSC experiments) were correlative and were successfully combined to evaluate the thermo‐stabilization of TR. Collectively, four of 10 TR mutants designed were confirmed to be thermo‐stabilized, where a thermo‐stabilized mutant was defined as a mutant showing a higher *T*_m_ by not less than 1°C when compared to the wild type.

**TABLE 2:** Experimental characterization of TR

Construct	Residual pigment (%)^a^	Relative yield^b^	Decoloration rate constant^c^	*T*_m_ (°C)^d^	*P*‐Value^e^
WT	44.5 ± 3.2	1.0	0.21 ± 0.01	91.78 ± 0.34	‐
S8D	21.7 ± 2.9	0.81	‐	‐	‐
V79K	61.4 ± 4.0	0.69	0.11 ± 0.01	94.52 ± 0.07	.0014
T114D	67.6 ± 3.2	1.3	0.069 ± 0.001	95.93 ± 0.04	.0060
A115P	66.5 ± 4.8	0.50	0.11 ± 0.01	94.37 ± 0.06	.0017
A116E	69.5 ± 6.3	0.96	0.11 ± 0.01	94.04 ± 0.08	.0029
T143K	49.1 ± 4.9	‐	‐	‐	‐
A177K	35.9 ± 2.7	0.66	‐	‐	‐
W210R	12.6 ± 1.0	0.74	‐	‐	‐
G214D	35.8 ± 5.0	0.95	‐	‐	‐
A215P	36.0 ± 4.5	1.2	‐	‐	‐

^a^ Residual pigment is calculated as “the maximum absorption at the wavelength 530 nm of TR after 4‐minutes incubation at 90 °C" divided by “that before incubation” and is represented in the percentage; the higher value indicates a thermo‐stabilization.

^b^ For a TR construct, the relative amount of purified sample when compared to that of the wild type is shown. The expression level of T143K was too low to purify it for further characterizations. The difference of the residual pigment between T143K and WT was not also significant, thereby regarding it as a destabilizing mutant.

^c^ The decoloration rate constants at 90°C; the lower value indicates a thermo‐stabilization.

^d^ Temperature at the top of the heat absorption peak from the DSC experiment. The higher value indicates a thermo‐stabilization.

^e^ *T*_m_ values from a mutant and from the wild type are compared. The Welchʼs *t*‐test was applied for the T114D mutant; the Studentʼs *t*‐test was applied for the others.

### 3.3 MD simulation of TR at 300 K

**FIGURE 3:**
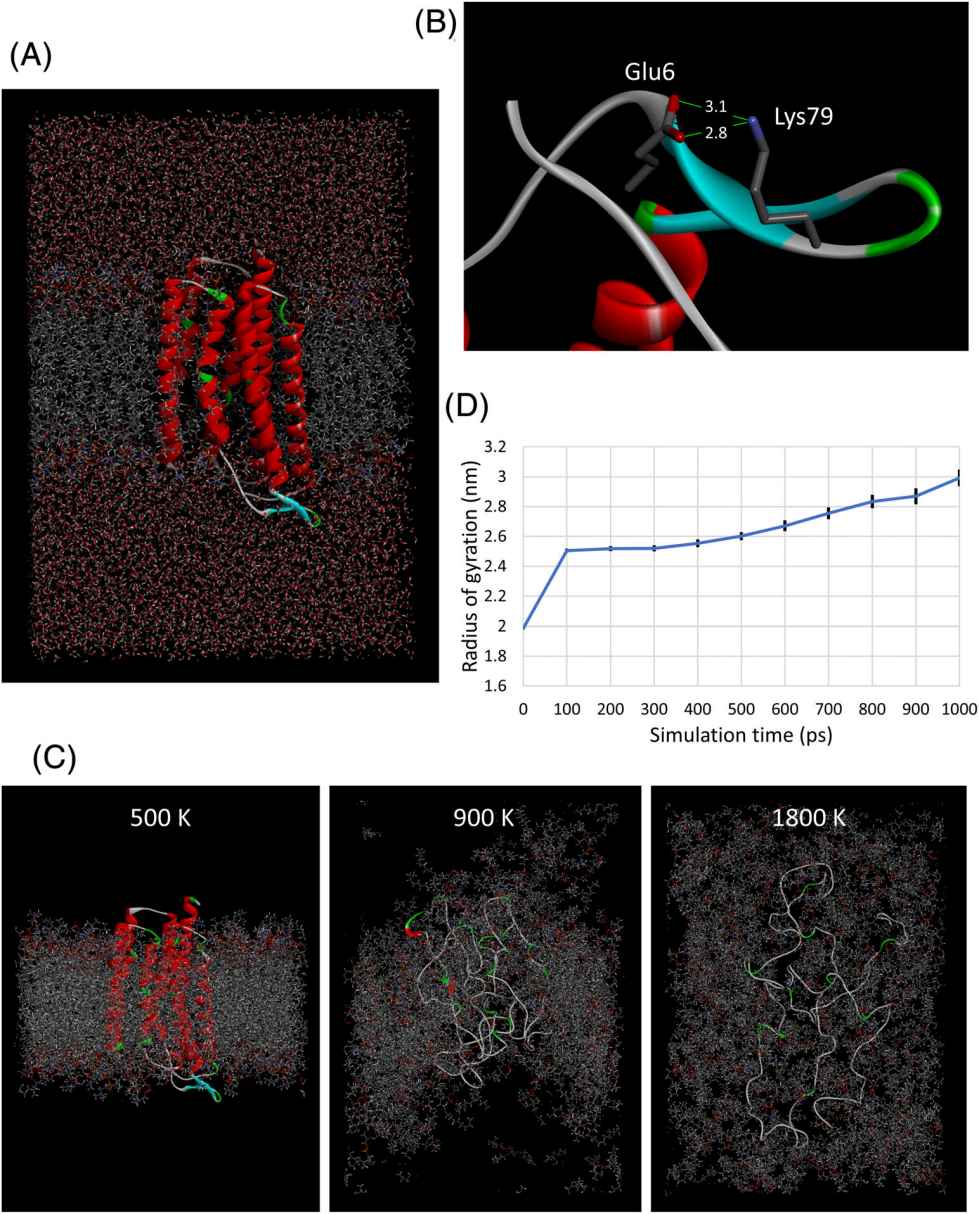
Analysis of TR structures using MD simulation. A, MD simulation of wild‐type TR in native state. A whole MD structure in a rectangular unit‐cell at 100 ns from an NPT simulation at 300 K is shown. The TR molecule is represented as a ribbon model with secondary‐structure colorings. The other molecules are depicted as stick models with atom‐type colorings. B, MD structure of V79K mutant. An MD structure at 100 ns from an NPT simulation at 300 K is shown. The TR molecule is represented as a ribbon model with secondary‐structure colorings. The side‐chains of Glu6 and Lys79 are depicted as stick models with atom‐type colorings and with interaction distances in Å. C, MD simulation of wild‐type TR at high temperatures. Whole MD structures in a rectangular unit‐cell at 1 ns from an NVT simulation at three different temperatures are shown. The TR molecule is represented as a ribbon model with secondary‐structure colorings. The membrane molecules are depicted as stick models with atom‐type colorings. The solvent molecules are not shown for clarity. The Figures A‐C, were produced using *Discovery Studio*. D, Radius of gyration in MD simulation at 1800 K. A time course of the radius of gyration for the MD simulation of the wild‐type TR at 1800 K is shown. The radius of gyration was calculated using the “gmx gyrate” function of *GROMACS*. Average values from 20 independent MD runs at every 100 ps in the MD simulation are plotted with error bars representing s.e.m. (*n* = 20) [Color figure can be viewed at wileyonlinelibrary.com]

It is intriguing to ask whether the hit rate of the present thermo‐stabilization design based on the free‐energy calculation can be improved or not by a subsequent evaluation of candidates using the MD simulation. To examine this possibility, first we evaluated the TR structures in the native state through an MD simulation at 300 K for 100 ns with an NPT periodic‐boundary model containing explicit membrane/solvent molecules (Figure 3A). The C^*α*^ superposition analysis of the MD structures with the crystal structure revealed a statistically significant structural difference of the most thermo‐stabilized T114D mutant and no significant differences for the other mutants when compared to the wild type (Table 3). This analysis calculates r.m.s.d. values from the C^*α*^ superpositions between the crystal structure and the native‐state MD structures to evaluate the degree of mutation‐induced structural changes that should be small in a successful design. It is noteworthy that the MD structures of V79K reveal an ion‐pair between Glu6 and Lys79 as it was designed (Figure 3B); 460 of 501 MD structures at 0‐100 ns (92%) have short Glu6 C^δ^ − Lys79 N^ζ^ distances not greater than 5 Å. To clarify the location of the mutation‐induced structural changes in the T114D mutant, the C^*α*^ deviations from the crystal structure were calculated by residue for the T114D mutant and compared with those for the wild type (Supplementary Table S1). As a result, 27 residues showed significantly larger deviations in the T114D mutant when compared to those in the wild type. These residues do not interact directly with the 114th residue, suggesting an indirect mode of the T114D‐induced structural change. The most significant structural changes are observed in the N‐terminal extramembrane region located about 50 Å away from the mutation site. In addition, moderate structural changes are observed in the region of residues 170 to 190 located on the same four‐helix bundle to which the 114th residue belongs. Thus, it is conceivable that the T114D‐induced structural change originates in the region of residues 170 to 190 located about 10 to 20 Å away from the mutation site and then propagates to the other regions with longer distances, although further clarification on the indirect mechanism was unsuccessful. Probably, the influence of the T114D mutation is not straightforward because it aims the stabilization of the helix dipole with broad directionality. We also analyzed the mutation‐induced structural changes in other nine mutants and found less remarkable structural changes when compared to those in the T114D mutant (Supplementary Table S1). It should be noted that the present superposition analysis for the native‐state MD structures uses the crystal structure as the reference, and therefore it does not reflect the structural dynamics within the MD structures. To further investigate the TR structures in the native state, the structural dynamics should be considered in future by introducing such as the principal component analysis^27^ and the cluster analysis.^28^

**TABLE 3:** MD simulation of TR in native state

Construct	R.m.s.d. (Å)^a^	*P*‐Value^b^
WT	1.06 ± 0.06	‐
S8D	1.03 ± 0.03	.61
V79K	1.29 ± 0.08	.087
T114D	1.47 ± 0.06	.0072
A115P	1.05 ± 0.08	.93
A116E	1.15 ± 0.15	.62
T143K	1.26 ± 0.14	.29
A177K	1.25 ± 0.11	.21
W210R	1.21 ± 0.08	.22
G214D	1.07 ± 0.06	.90
A215P	1.11 ± 0.09	.70

^a^ Average value from C^*α*^ superpositions of MD structures with the crystal structure. For a construct, r.m.s.d values from C^*α*^ superpositions of 51 MD structures at 90−100 ns with the crystal structure are averaged in an MD run, and an average of the average r.m.s.d. values from three independent MD runs is shown with its s.e.m. (*n* = 4 for T143K; *n* = 3 for the others).

^b^ Values from a mutant and from the wild type are compared.

### 3.4 MD simulation of TR at high temperatures

To evaluate the TR structures in the denatured state, we tried MD simulations at high temperatures for 1 ns using an NVT periodic‐boundary model with explicit membrane/solvent molecules after an NPT equilibration of the system at 300 K. First, appropriate MD conditions were searched by changing temperature in the range of 300 to 2000 K (Figure 3C; Supplementary Figure S2). At temperatures in the range of 300 to 500 K, r.m.s.d. values from C^*α*^ superpositions between the MD structures and the crystal structure were less than 2 Å, and no deformation of the membrane was observed, indicating that the native structure was kept at these temperatures for 1 ns. At temperatures in the range of 600 to 800 K, the r.m.s.d. values increased to the range of 2 to 7 Å, and clear deformations of the membrane were observed, indicating a partial denaturation of protein/membrane. At temperatures in the range of 900 to 1600 K, the r.m.s.d. values jumped to the range of 12 to 18 Å probably due to the collapse of helical structures, and the membrane structure was partially broken. At temperatures in the range of 1700 to 2000 K, all secondary structures except for turns disappeared with r.m.s.d. values in the range of 20 to 25 Å, and the membrane structure was completely broken with a homogeneous distribution of lipid molecules. Considering these results, an NVT simulation for 1 ns at 1800 K was adopted in this work to evaluate the TR structures in the denatured state. Although this condition did not represent a thermodynamic equilibrium of the system, the reproducibility of the simulation was confirmed by 20 independent runs for each TR construct.

To evaluate the compactness of the TR molecule in the MD simulation at 1 800 K, the radius of gyration was calculated for the wild‐type TR (Figure 3D). When compared to the radius of gyration of the crystal structure of about 2.0 nm, that of the MD structure after 1 ns increased by 1.5‐fold to be about 3.0 nm. This coincides with a reported experimental transition in the radius of gyration between the native and the random‐coil states of apomyoglobin from a small‐angle X‐ray scattering study,^29^ whereas, this MD structure is clearly different from that of a partially unfolded “molten‐globule state”^30^ with retaining secondary structures. Thus, the MD simulation of TR for 1 ns at 1800 K may provide a random‐coil structure. Importantly, this random‐coil TR molecule is highly reproducible in the simulation, and it does not be extended completely, indicating that long‐range intramolecular interactions still present in this condition.

According to the Boltzmann's principle, the entropy of a system increases by increasing the number of microscopic states of the system, indicating that an increment in the intramolecular structural fluctuation of a random‐coil protein molecule means an increment of entropy in the denatured state, thereby destabilizing the protein folding. In the present study, average r.m.s.d. values from C^*α*^ superpositions of MD structures from 20 independent NVT simulations for 1 ns at 1800 K were used to evaluate the structural variation/fluctuation of TR in the random‐coil state. It should be noted that the reference structure used for this r.m.s.d. calculation is a representative MD structure so as to give the smallest average r.m.s.d. value from superpositions to the other 19 MD structures, which is relevant to the procedure used for the cluster analysis on the MD structures of Keap1 protein.^28^ Therefore, this r.m.s.d. analysis within the 20 MD structures may reflect the variation of structures (ie, entropy) in the random‐coil state. The superposition analysis of the MD structures revealed a statistically significant structural variation of the S8D mutant when compared to the wild type and no differences for the other mutants (Table 4). It is conceivable that the S8D mutation induces a larger structural fluctuation in the random‐coil state when compared to the wild type and therefore it entropically destabilizes the folding. Unfortunately, the TR molecules in the random‐coil state were too mobile to rationalize the larger structural fluctuation of the S8D mutant. Although the structural variation of TR constructs in the random‐coil state was analyzed in this work by adopting a drastic NVT simulation for 1 ns at 1 800 K, longer NPT simulations at lower temperatures should be considered in future to investigate further on the denatured state of TR.

**TABLE 4:** MD simulation of TR in random‐coil state

Construct	R.m.s.d. (Å)^a^	*P*‐Value^b^
WT	22.4 ± 0.5	‐
S8D	24.9 ± 0.4	.00050
V79K	23.1 ± 0.7	.42
T114D	22.7 ± 0.5	.69
A115P	22.7 ± 0.6	.69
A116E	23.3 ± 0.6	.26
T143K	22.3 ± 0.6	.87
A177K	22.7 ± 0.6	.76
W210R	22.9 ± 0.4	.45
G214D	23.3 ± 0.6	.27
A215P	22.7 ± 0.4	.70

^a^ Average value with its s.e.m. (*n* = 19) from C^*α*^ superpositions of MD structures.

^b^ Values from a mutant and from the wild type are compared.

## 4 DISCUSSION

Previously, we reported a hyperthermostabilization design of a soluble globular protein, CutA1, which was based on the computational prediction of folding free‐energy differences upon mutation.^11^ In the present study, we applied the same strategy to a thermostable membrane protein, TR. Here we designed 10 mutants in the extramembrane regions of TR, and four of them were found to be stabilized, indicating a hit‐rate of 40%. This value is comparable to 39% in the case of CutA1.^31^ Therefore, introducing a single mutation in the extramembrane regions based on the folding free‐energy prediction may be useful to design a thermo‐stabilized TR mutant. In the next step, the four successful single mutations obtained here will be used to design a further thermo‐stabilized TR mutant with multiple mutations. It should be noted that the predicted changes in free energy for the mutants (Table 1) do not necessarily translate to the magnitude of changes measured experimentally (Table 2), indicating that the “Build Model” function of *FoldX* may overestimate the free‐energy changes. Other MD‐based design methods such as the free‐energy perturbation calculation^32^ may be examined in future to compare results. For practical applications such as optogenetics tools and biosensors, the functional activity of the mutants should be evaluated carefully.

In this study, we examined the possibility of an improvement of the thermo‐stabilization design using the MD simulation. Although the MD simulation in the native state could not improve the hit rate of the design, it suggested a mutation‐induced structural change of the T114D mutant that revealed the highest *T*_m_ in the DSC experiment. Thus, the T114D mutation is thermo‐stabilizing but may induce a structural change in the native state, although it is unclear whether the mutation‐induced structural change contributes to the thermo‐stabilization or not. This result indicates that the MD simulation in the native state may suggest a mutation‐induced structural change that can affect the protein function. On the other hand, the MD simulation in the random‐coil state could improve the hit rate by detecting an entropic destabilization of the S8D mutant that revealed the lower thermostability than that of the wild type. Thus, in certain cases, the MD simulation may suggest candidates with problems in the denatured state that is not considered explicitly in the folding free‐energy prediction.

In conclusion, the present thermo‐stabilization design in the extramembrane regions based on the free‐energy calculation and the subsequent evaluation by the MD simulation may be useful to improve the production of membrane proteins. The engineering in the intramembrane region.^7^, ^8^ would be a complementary design method. These two strategies in the extra‐ and intramembrane regions will be combined in future to design hyperthermostable mutants of TR tolerable at temperatures over 100°C. Further applications of the strategies to other membrane proteins may expand the availability of the rational design of proteins in general.

## CONFLICT OF INTEREST

The authors have no conflict of interest to report.

5

### PEER REVIEW

The peer review history for this article is available at https://publons.com/publon/10.1002/prot.26015.

## Supporting information


**Appendix** **S1:** Supplementary Information
