# Supplementary material for: Further thermo‐stabilization of thermophilic rhodopsin from Thermus thermophilus JL‐18 through engineering in extramembrane regions
Source: Proteins. 2020 Oct 28;89(3):301–10. doi: 10.1002/prot.26015 (PMC7894484; doi:10.1002/prot.26015)
Supplement: Supplementary file 1 — Appendix S1: Supplementary Information [file PROT-89-301-s001.pdf]

Supporting information for "Further thermo-stabilization of thermophilic rhodopsin from *Thermus thermophilus* JL-18 through engineering in extramembrane regions"

Tomoki Akiyama[3], Naoki Kunishima[1,2*], Sayaka Nemoto[3], Kazuki Kazama[3], Masako Hirose[4], Yuki Sudo[5], Yoshinori Matsuura[2], Hisashi Naitow[2], Takeshi Murata[3*]

[1]RIKEN RSC-Rigaku Collaboration Center, [2]RIKEN SPring-8 Center, 1-1-1 Kouto, Sayo-cho, Sayo-gun, Hyogo 679-5148, Japan.

[3]Department of Chemistry, Graduate School of Science, and Molecular Chirality Research, Chiba University, 1-33 Yayoi-cho, Inage-ku, Chiba-shi, Chiba 263-8522, Japan.

[4]Malvern Panalytical division of Spectris Co., Ltd., 1-7-3 Hamamatsu-cho, Minato-ku, Tokyo 105-0013, Japan.

[5]Division of Pharmaceutical Sciences, Graduate School of Medicine, Dentistry, and Pharmaceutical Sciences, Okayama University, Okayama 700-8530, Japan.

[*]e-mail: naoki.kunishima@alum.riken.jp; t.murata@faculty.chiba-u.jp

Correspondence and requests for materials should be addressed to N.K. or T.M.

Contents:

Supplementary Figure S1. Detail of DSC experiment on wild-type TR.

Supplementary Figure S2. MD simulation of wild-type TR at high temperatures.

Supplementary Table S1. Mutation-induced structural changes in native-state MD.

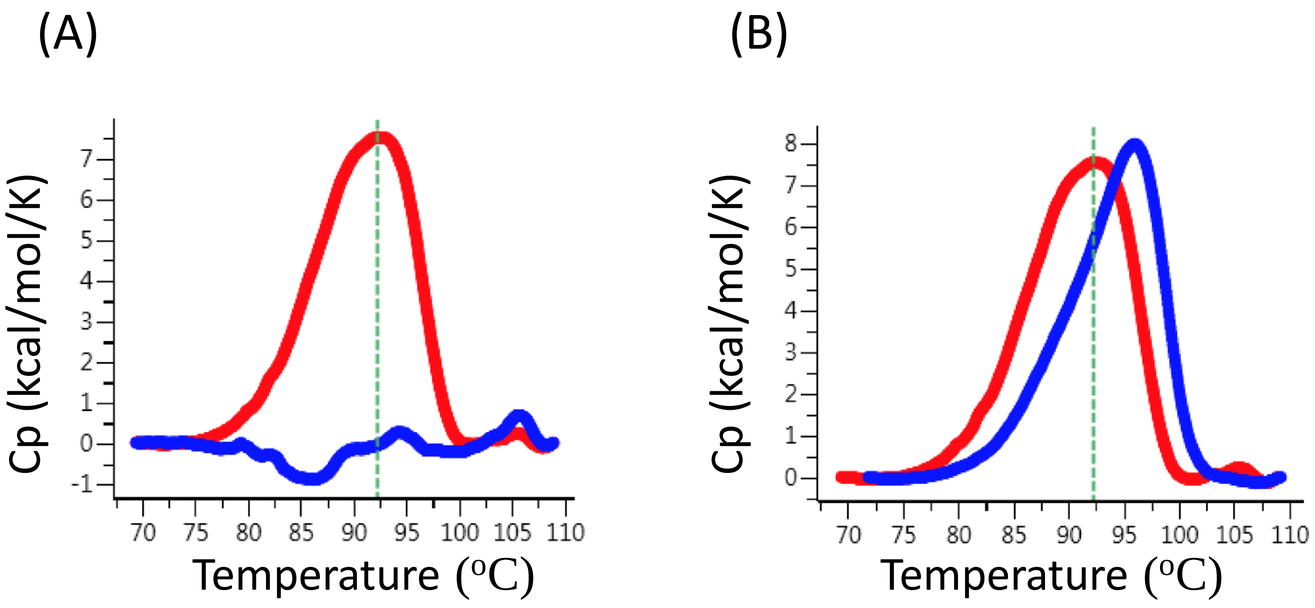

FIGURE S1 Detail of DSC experiment on wild-type TR. (A) DSC rescan experiment. Red line: first scan. Blue line: second scan of the scanned sample. (B) DSC experiments at different scan rates. Red line: 60 ºC/hr (default rate). Blue line: 120 ºC/hr. These data suggest that the TR denaturation is an irreversible/kinetic process

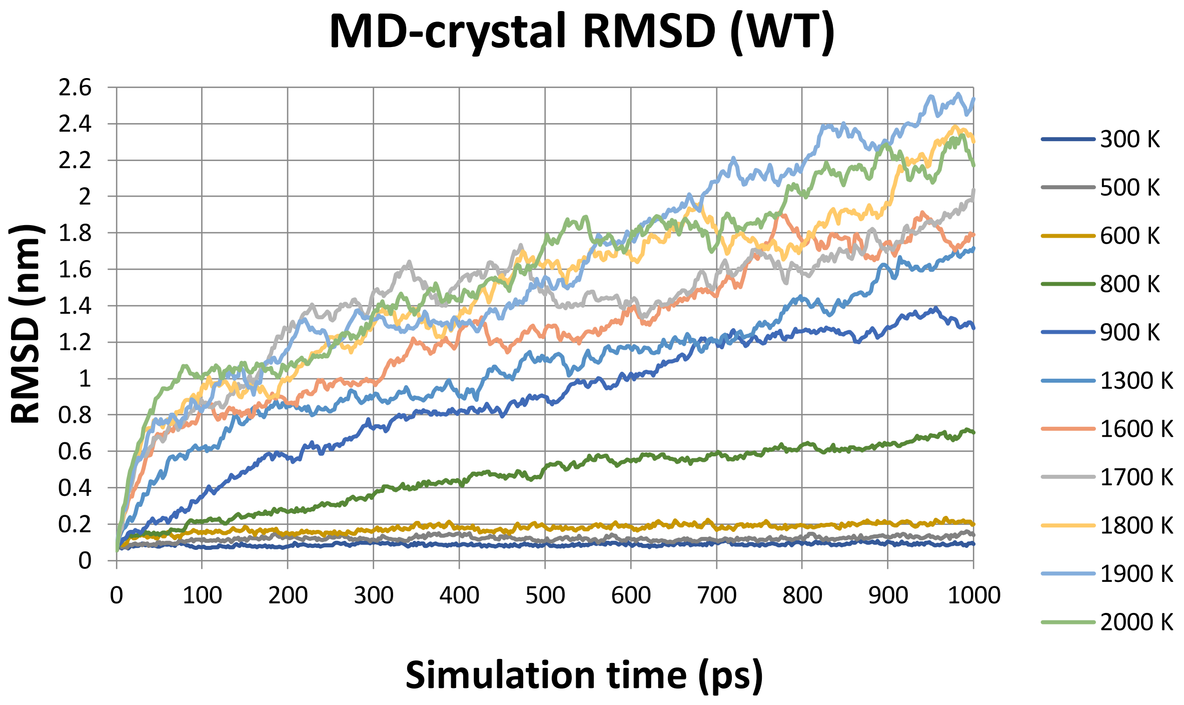

FIGURE S2 MD simulation of wild-type TR at high temperatures. To determine an appropriate condition for the MD simulation in the random-coil state, the wild-type TR was submitted to NVT runs for 1 ns at various temperatures in the range of 300–2000 K. For each run at a specific temperature, r.m.s.d. values from C^{α} superpositions between the MD structures and the crystal structure are plotted as a time-course

TABLE S1 Mutation-induced structural changes in native-state MD

Residue number	Average C^{α} deviation (Å)[a]		*p*-Value[b]	Distance (Å)[c]
	mutant	WT		
	S8D			
196	0.52 ± 0.02	0.67 ± 0.03	0.01632	30.2
88	1.74 ± 0.25	2.68 ± 0.04	0.02037	19.5
188	0.74 ± 0.04	0.58 ± 0.02	0.02455	41.8
230	0.44 ± 0.03	0.63 ± 0.05	0.03607	25.5
	V79K			
111	0.77 ± 0.02	0.59 ± 0.02	0.00350	51.4
157	1.22 ± 0.09	0.76 ± 0.07	0.01449	43.3
112	1.48 ± 0.18	0.73 ± 0.09	0.02051	54.4
81	1.29 ± 0.07	0.89 ± 0.09	0.02238	6.0
194	0.67 ± 0.05	0.48 ± 0.02	0.02751	37.8
40	1.05 ± 0.06	0.73 ± 0.09	0.04300	50.6
248	1.01 ± 0.10	0.68 ± 0.06	0.04591	54.4
	T114D			
6	2.41 ± 0.08	1.18 ± 0.14	0.00168	55.1
189	0.99 ± 0.04	0.63 ± 0.05	0.00381	22.0
172	1.34 ± 0.09	0.73 ± 0.06	0.00432	12.5
170	0.86 ± 0.06	0.54 ± 0.03	0.00865	12.1
14	1.40 ± 0.09	0.92 ± 0.05	0.0104	46.8
138	1.47 ± 0.09	0.88 ± 0.11	0.0144	36.2
171	1.29 ± 0.13	0.75 ± 0.05	0.0176	13.9
214	4.30 ± 0.61	1.41 ± 0.44	0.0184	50.8
190	0.80 ± 0.03	0.53 ± 0.06	0.0187	20.1
151	1.17 ± 0.13	0.68 ± 0.02	0.0201	35.8
174	1.23 ± 0.12	0.75 ± 0.04	0.0204	10.5
143	1.83 ± 0.15	1.13 ± 0.12	0.0222	45.7
173	0.99 ± 0.11	0.64 ± 0.03	0.0306	8.8
5	2.84 ± 0.48	1.12 ± 0.22	0.0311	52.6
155	0.77 ± 0.04	0.58 ± 0.04	0.0312	30.0
212	2.13 ± 0.11	1.65 ± 0.11	0.0329	50.3
139	1.22 ± 0.16	0.68 ± 0.08	0.0388	37.5
15	1.13 ± 0.08	0.68 ± 0.12	0.0395	44.6
74	1.46 ± 0.18	0.87 ± 0.08	0.0413	55.4
70	1.96 ± 0.24	1.11 ± 0.16	0.0423	48.2
238	0.64 ± 0.04	0.47 ± 0.04	0.0427	23.2
218	2.27 ± 0.36	1.11 ± 0.17	0.0442	45.6
215	2.73 ± 0.51	1.04 ± 0.29	0.0443	50.6
150	1.65 ± 0.26	0.88 ± 0.05	0.0452	38.4
163	0.66 ± 0.07	0.46 ± 0.02	0.0479	20.8
188	0.73 ± 0.05	0.58 ± 0.02	0.0488	19.7
175	1.65 ± 0.28	0.85 ± 0.07	0.0498	12.6
	A115P			
88	2.38 ± 0.09	2.68 ± 0.04	0.03985	40.8
10	1.20 ± 0.04	0.94 ± 0.08	0.04678	52.2
147	1.35 ± 0.17	0.87 ± 0.03	0.04824	40.9
	A116E			
153	0.68 ± 0.03	0.95 ± 0.05	0.01156	31.6
197	0.70 ± 0.02	0.58 ± 0.03	0.02324	29.0
189	1.03 ± 0.11	0.63 ± 0.05	0.02934	24.9
213	3.03 ± 0.33	2.00 ± 0.13	0.04454	48.4
	T143K			
160	1.01 ± 0.10	0.53 ± 0.03	0.01071	24.8
142	1.96 ± 0.14	1.25 ± 0.17	0.02344	3.8
197	0.90 ± 0.09	0.58 ± 0.03	0.03047	25.3
193	0.86 ± 0.09	0.52 ± 0.04	0.03225	31.2
39	0.77 ± 0.07	1.22 ± 0.17	0.04019	48.9
	A177K			
197	0.90 ± 0.02	0.58 ± 0.03	0.00125	27.8
217	2.59 ± 0.15	1.12 ± 0.24	0.00631	46.5
153	0.68 ± 0.02	0.95 ± 0.05	0.00956	34.9
177	1.31 ± 0.11	0.76 ± 0.10	0.01824	0.0
218	2.24 ± 0.28	1.11 ± 0.17	0.02665	46.5
4	2.89 ± 0.44	1.20 ± 0.29	0.03256	53.2
193	0.68 ± 0.03	0.52 ± 0.04	0.03925	23.1
175	1.37 ± 0.16	0.85 ± 0.07	0.04146	5.5
111	0.80 ± 0.07	0.59 ± 0.02	0.04486	9.9
112	1.10 ± 0.10	0.73 ± 0.09	0.04878	9.2
151	0.87 ± 0.06	0.68 ± 0.02	0.04935	37.9
	W210R			
218	2.08 ± 0.15	1.11 ± 0.17	0.01291	13.2
156	0.69 ± 0.02	0.60 ± 0.02	0.02232	18.0
193	0.67 ± 0.02	0.52 ± 0.04	0.02602	26.8
214	2.89 ± 0.09	1.41 ± 0.44	0.03039	11.6
63	0.72 ± 0.02	1.08 ± 0.11	0.03321	28.1
64	0.80 ± 0.03	1.04 ± 0.07	0.03442	26.6
244	0.52 ± 0.01	0.66 ± 0.05	0.03983	42.1
209	1.45 ± 0.17	0.84 ± 0.12	0.04327	3.8
215	2.36 ± 0.02	1.04 ± 0.29	0.04509	14.7
152	0.79 ± 0.09	0.50 ± 0.04	0.04991	13.2
	G214D			
150	1.37 ± 0.08	0.88 ± 0.05	0.00737	24.6
151	0.87 ± 0.04	0.68 ± 0.02	0.01471	24.4
189	0.86 ± 0.03	0.63 ± 0.05	0.01472	35.1
188	0.79 ± 0.05	0.58 ± 0.02	0.02139	37.6
153	0.67 ± 0.06	0.95 ± 0.05	0.02410	25.5
174	0.99 ± 0.07	0.75 ± 0.04	0.04304	46.4
165	0.56 ± 0.03	0.44 ± 0.03	0.04924	38.0
	A215P			
147	1.26 ± 0.09	0.87 ± 0.03	0.01453	24.8
150	1.12 ± 0.03	0.88 ± 0.05	0.01463	27.1
169	0.55 ± 0.02	0.612 ± 0.004	0.02705	43.0
153	0.71 ± 0.05	0.95 ± 0.05	0.03314	27.6
68	0.70 ± 0.05	1.00 ± 0.08	0.03325	16.5
194	0.56 ± 0.02	0.48 ± 0.02	0.03839	28.7
65	0.68 ± 0.03	0.93 ± 0.08	0.04155	17.2
67	0.77 ± 0.06	1.00 ± 0.05	0.04945	18.7

[a]Values from C^{α} superpositions between the crystal structure and the MD structures from the native-state NPT simulations. For each residue in a construct, deviation values from C^{α} superpositions of 51 MD structures at 90−100 ns with the crystal structure are averaged in an MD run, and an average of the average C^{α} deviation values from three or four independent MD runs is shown with its s.e.m. (n = 4 for the T143K mutant and n = 3 for the others).

[b]The average C^{α} deviation value of a mutant and that of the wild type are compared. This table is sorted by the *p*-value in ascending order.

[c]C^{α}−C^{α} interatomic distances in the crystal structure between the mutation residue and the residue of noted. The results for all ten mutants are shown.
